# Does surgeon or hospital volume influence outcome in dedicated colorectal units?—A Viennese perspective

**DOI:** 10.1007/s00508-024-02405-6

**Published:** 2024-08-02

**Authors:** Gabor J. Schuld, Lukas Schlager, Matthias Monschein, Stefan Riss, Michael Bergmann, Peter Razek, Anton Stift, Lukas W. Unger

**Affiliations:** 1 Division of Visceral Surgery, Dept. of General Surgery, Medical University of Vienna, Medical University of Vienna, Währinger Gürtel 18–20, 1090 Vienna, Austria; 2 Hospital Floridsdorf, Department of General Surgery, Brünner Straße 68, 1221 Vienna, Austria

**Keywords:** Colorectal surgery, Hospital volume, Surgical expertise, Postoperative complications, Colorectal cancer

## Abstract

**Objective:**

A clear relationship between higher surgeon volume and improved outcomes has not been convincingly established in rectal cancer surgery. The aim of this study was to evaluate the impact of individual surgeon’s caseload and hospital volume on perioperative outcome.

**Methods:**

We retrospectively analyzed 336 consecutive patients undergoing oncological resection for rectal cancer at two Viennese hospitals between 1 January 2015 and 31 December 2020. The effect of baseline characteristics as well as surgeons’ caseloads (low volume: 0–5 cases per year, high volume > 5 cases per year) on postoperative complication rates (Clavien-Dindo Classification groups of < 3 and ≥ 3) were evaluated.

**Results:**

No differences in baseline characteristics were found between centers in terms of sex, smoking status, or comorbidities of patients. Interestingly, only 14.7% of surgeons met the criteria to be classified as high-volume surgeons, while accounting for 66.3% of all operations. There was a significant difference in outcomes depending on the treating center in univariate and multivariate binary logistic regression analysis (odds ratio (OR) = 2.403, *p* = 0.008). Open surgery was associated with lower complication rates than minimally invasive approaches in univariate analysis (OR = 0.417, *p* = 0.003, 95%CI = 0.232–0.739) but not multivariate analysis. This indicated that the center’s policy rather than surgeon volume or mode of surgery impact on postoperative outcomes.

**Conclusion:**

Treating center standards impacted on outcome, while individual caseload of surgeons or mode of surgery did not independently affect complication rates in this analysis. The majority of rectal cancer resections are performed by a small number of surgeons in Viennese hospitals.

**Supplementary Information:**

The online version of this article (10.1007/s00508-024-02405-6) contains supplementary material, which is available to authorized users.

## Introduction

Colorectal cancer (CRC) remains the third most commonly diagnosed cancer worldwide, accounting for approximately 1.9 million new cases annually [1]. Despite the implementation of screening programs, a better understanding of the underlying molecular mechanisms and individualized treatment algorithms defined in multidisciplinary team meetings, CRC still accounts for approximately 9.2% or more than 800,000 cancer-related deaths per year globally, ranking it the second most deadly cancer after lung malignancies [2]. Surgery is the treatment of choice for colon cancer without metastases or infiltration of adjacent organs as well as for locally advanced rectal cancer after neoadjuvant treatment. Both neoadjuvant treatment and surgery for rectal cancer are associated with a risk of severe complications for patients, partly due to the patient’s baseline characteristics and partly due to technical challenges within the often narrow pelvis [3]. As most patients diagnosed with CRC are older than 70 years of age [4] and often suffer from concomitant comorbidities [5–7], individualized treatment and limiting surgical trauma is of utmost importance. Existing guidelines and standardized training for surgeons aim to reduce rates of postoperative complications but surgical practices still vary greatly in terms of surgeon’s caseload and center’s policy. While in some countries subspecialization within general surgery or even colorectal surgery is common, general surgeon’s in Austria still cover a wider practice than in other countries, albeit subspecialization is common in bigger units. To compare the effects of this practice and benchmark internationally, a more detailed evaluation and comparison of case counts and performance between hospitals and surgeons are necessary but not readily available; however, a multitude of studies, mainly from North America but also a meta-analysis including several European countries and Japan, underscored the correlation between hospital and surgeon volumes and patient outcomes. This was shown for malignancies such as hepatobiliary and pancreatic [8], thoracic [9], esophageal [10], and urological cancers [11]. Studies in CRC are inconclusive and do not clearly demonstrate an advantage for high-volume centers [12–15].

Although the adoption of general concepts such as total mesorectal excision (TME) are universally accepted [16], implementation of different enhanced recovery after surgery bundles and utilization of minimally invasive techniques vary between units. Public healthcare is provided by several large district general hospitals (DGH) as well as a university clinic in addition to several smaller privately run but publicly accessible providers in Vienna, and the two analyzed units are the biggest units offering a dedicated colorectal service. Staffing levels between hospitals analyzed in this study are comparable as the vast majority of the staff are employed by the same employer but doctors in the university clinic are employed by the Medical University of Vienna. In recent years, centralization and subsequently the establishment of high-volume centers has been thought to aid proficiency and ultimately improve postoperative outcomes [17]; however, the CRC population has not been examined in this respect in Austria and concrete criteria and outcome parameters to clearly define and examine high-volume centers and high-volume surgeons are currently missing for colorectal surgery. There is no European consensus but analyses have shown that a cut-off of 5 rectal cancer resections per year is an acceptable cut-off to differentiate between low-volume and high-volume surgeons [18]. In Vienna, few data are available on individual surgeon’s performance, especially in relation to postoperative outcomes. Hence, we investigated the impact of surgeon’s caseload on perioperative complication rates, analyzing data of two specialized centers in Vienna.

## Material and methods

### Data source and study population

Data were collected from electronic health records from two centers between 1 January 2015 and 31 December 2020. Overall, data from 471 consecutive patients undergoing surgery due to rectal cancer at the Medical University of Vienna and the KH Floridsdorf were evaluated. Inclusion criteria were ≥ 18 years of age and histologically verified diagnosis of rectal cancer. Exclusion criteria were colonic cancer locations other than rectal cancer, emergency surgery, surgery performed by trainees and data completeness < 90%.

A total of 336 patients met the criteria and were included in the analyses. Data were subjected to quality control by two additional independent researchers and subsequently analyzed. The collected variables for every patient included sex, neoadjuvant (chemo)radiotherapy, tumor location, TNM stage, smoking habits at the time of surgery and comorbidity burden, summarized by the Charlson comorbidity index. For perioperative characteristics we assessed operating surgeon’s case volume per year and the mode of surgery (minimally invasive or open).

### Outcome measures

To assess the postoperative outcome we utilized the Clavien-Dindo Classification (CDC) [19], a widely used classification system for postoperative complications. The CDC differentiates five severity grades for postoperative complications, from “any deviation from a normal postoperative course” to “death”. The CDC was dichotomized for statistical analysis with a CDC of 0-2 being classified as a minor complication and a CDC of 3-5 as major complication, reflecting the clinical relevancy of minor complications not needing any invasive procedures and major complications needing invasive procedures for treatment. 

### Surgeon volume variable

Surgeons were divided into two groups based on their surgical volumes: low (0–5 cases per year), and high (> 5 cases per year), calculated as mean volume per year over the observed period, as indicated by international guidelines [20].

### Statistical analysis

Statistical analysis was performed using SPSS (Version 29.0.0.0 (241), IBM, Armonk, New York, United States). Distribution of baseline characteristics was compared between groups using χ^2^ or t‑test, as appropriate. Univariate and multivariate binary logistic regression was conducted in order to determine whether surgeon volumes impact on postoperative outcomes. The other variables tested for their impact on postoperative outcomes, such as neoadjuvant therapy [21], CCI as a measure for comorbidities [22], and type of surgery [23] were selected due to previous studies showing a possible influence on postoperative outcomes. A *p*-value < 0.05 was considered to indicate statistical significance.

### Data visualization

Data were visualized using GraphPad Prism (Version 9.5.1 (528), GraphPad Software, Inc.).

## Results

### Lower complication rates in open resections

**Fig. 1:**
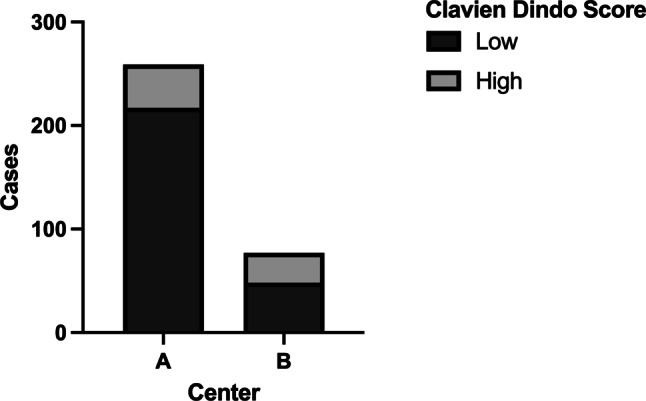
Percentages of low Clavien-Dindo Classification (CDC; 0–2) and high CDC (> 2) for the two centers demonstrate a significant difference (χ^2^-test *p* < 0.001) between centers

To first address center effects, we investigated the relationship between hospital volume with the postoperative outcome of patients. Overall 336 patients from the 2 centers met the inclusion criteria of the study, with 259 (77.1%) having undergone surgery in Center A and 77 (22.9%) in Center B. Examination of the postoperative complications, as assessed by the CDC, showed that 83.8% of patients undergoing a rectal resection in Center A and 62.3% of patients in Center B showed no major postoperative complications (Fig. 1, *p* < 0.001). These differences were not attributable to a difference in baseline characteristics, as sex, active smoking status, and comorbidities as assessed by the CCI were similar between centers (Supplemental Table 1).

**Fig. 2:**
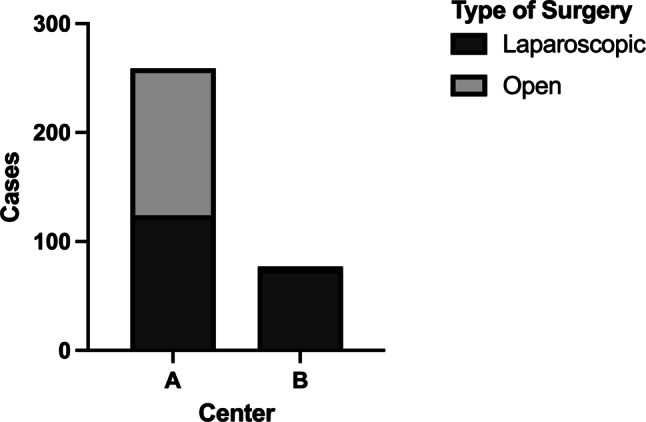
Distribution of utilization of minimally invasive surgery between centers (χ^2^-test *p* < 0.001)

**Fig. 3:**
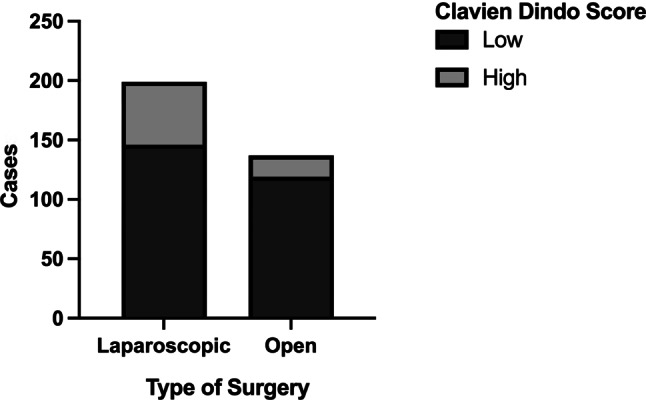
Frequency of complications in minimally invasive and open rectal cancer resections (χ^2^ test *p* = 0.003)

The occurrence of complications was inversely correlated to utilization of minimally invasive techniques, with Center B utilizing minimally invasive surgery more frequently (Fig. 2, *p* < 0.001). Center A had an approximately equal distribution between minimally invasive (124 cases) and open (135 cases) surgery, whereas more than 94% of surgeries in Center B were minimally invasive procedures; however, when analyzing both centers combined, minimally invasive surgery was associated with higher complication rates than open surgery (Fig. 3; *p* = 0.003). Ultimately, we performed univariate analysis to evaluate the risk for complications for several variables. Univariate binary logistic regression analysis showed that patients operated on at Center B had a higher risk for severe postoperative complications (odds ratio, OR = 3.122, *p* < 0.001) and that open surgery was associated with improved postoperative outcome (OR = 0.417, *p* = 0.003). Importantly, the effect of the center on postoperative outcome remained an independent risk factor for complications while the mode of surgery did not in multivariable binary logistic regression analysis (Table 1).

**Table 1:** Logistic regression analyses evaluating impact of distinct variables on postoperative complications

	Univariate logistic regression				Multivariate logistic regression			
Variable	OR	95% CI		p‑Value	OR	95% CI		p‑Value
		Lower	Upper			Lower	Upper	
Center (Center B vs. Center A)	3.122	1.77	5.504	*<* *0.001*	2.403	1.263	4.573	*0.008*
Surgeon volume (high vs. low)	1.387	0.78	2.466	0.265	1.296	0.712	2.359	0.397
Neoadjuvant therapy (yes vs. no)	1.422	0.827	2.444	0.203	1.459	0.83	2.565	0.189
OP type (open vs. minimally invasive)	0.417	0.232	0.749	*0.003*	0.563	0.286	1.109	0.097
CCI (high vs. low)	1.495	0.883	2.531	0.134	1.658	0.951	2.89	0.075

Evaluated variables were center (Center A or Center B), surgeon volume (low or high volume), neoadjuvant therapy (yes or no), OP type (minimally invasive or open), and CCI (high or low). Univariate logistic regression identified a significant association between Center (Center A) and open surgery for improved postoperative outcomes. The association between center and a better postoperative outcome also translated to the multivariate analysis, while the relationship between type of surgery and postoperative outcome narrowly did not. The other variables did not show any significant association, although the CCI was close to significance with the multivariate analysis.

*OR* odds ratio, *CI* confidence interval, *CCI* Charlson comorbidity index, OP

### Higher surgeon volume does not improve postoperative outcomes

**Fig. 4:**
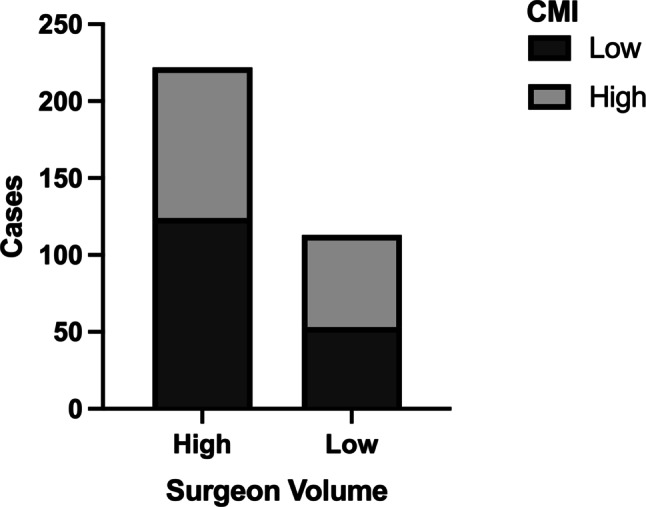
Relationship of surgeon volumes (low volume ≤ 5 cases per year, high volume > 5 cases per year) and Charlson comorbidity index (CCI, χ^2^ test *p* = 0.121)

**Fig. 5:**
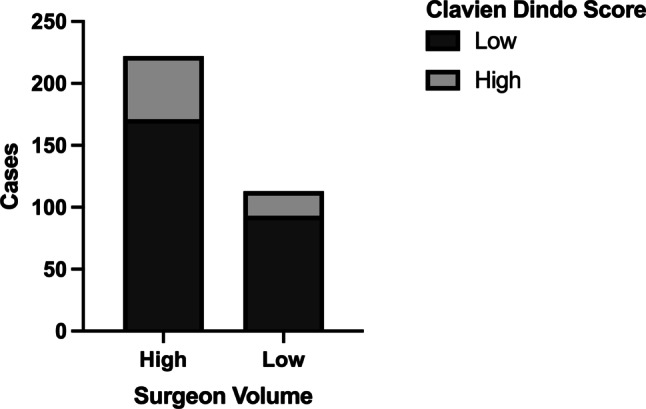
Surgeon volume (low volume ≤ 5 cases per year, high volume > 5 cases per year) and postoperative outcome, indicated by the Clavien-Dindo Classification (CDC) (*p* = 0.264)

While we identified a center effect to impact on the risk of complications, we additionally assessed differences in outcomes depending on surgeon’s case volume. We found that only 14.7% of surgeons met the criteria to be classified as high-volume surgeons but performed 66.3% of all operations. Thus, we further examined whether high-volume surgeons operated on patients with a higher comorbidity burden, as assessed by CCI, and found that there was no correlation between either low or high surgeon volume and low or high CCI (Fig. 4, *p* = 0.121). Equally, surgeon volume did not impact on postoperative complication rates (Fig. 5; *p* = 0.264), and surgeon case volume did not influence outcomes in univariate or multivariate binary logistic regression analysis (Table 1). The results therefore indicate that center effects other than individual surgeon’s performance are equally crucial for excellent results.

## Discussion

Our study aimed to investigate the relationship between surgeon volume and the postoperative outcomes of patients undergoing surgery for colorectal malignancies in two dedicated units in Vienna. By addressing this relationship, we sought to contribute further evidence to the ongoing debate regarding subspecialization within general surgery, and establishment of larger centers for CRC.

Consistent with previous research, our findings support a better postoperative outcome of patients in the higher volume hospital compared to the lower volume hospital, irrespective of the surgeon. Specifically, the higher volume hospital exhibited little or no postoperative complications in 83.8% of cases, whereas the lower volume hospital achieved this outcome in only 62.3% of cases; however, we found no significant association between surgeon volume and postoperative outcomes of patients. The more experienced surgeons did not primarily operate on more morbid patients, highlighting the role of hospital volume as the only significant predictor of postoperative outcomes of patients undergoing CRC surgery. While previous studies and the clinical context show that the individual surgical expertise is without a doubt crucial for patient outcomes [12, 24], our results indicate that the individual surgical expertise cannot function in isolation of the institutional framework in which it operates. Yet, regionalization of colorectal surgery could have compounding effects on the surgeon volume variable as well, which, as mentioned before, has been shown to be linked to improved postoperative outcomes [9, 25].

Importantly, we do not have specific data on 1) learning curves of individual surgeons or 2) compliance with the Enhanced Recovery After Surgery (ERAS) guidelines for individual patients in the analyzed cohort, as these data are not routinely assessed and difficult to investigate retrospectively, highlighting the need for further prospective observational data generation to improve service provision. Another noteworthy finding was the inverse relationship between worse postoperative outcomes and the higher rates of minimally invasive surgery at Center A, with the less invasive surgery also being associated with a higher risk for complications in univariate logistic regression analysis; however, this effect was not demonstrable in multivariate analysis including known risk factors. Center A conducted significantly less minimally invasive surgeries, while still having the better postoperative outcomes, even though laparoscopic surgery has been associated with better short-term outcomes after left-sided colon cancer resections [23]. This seemingly counterintuitive observation may be attributed to two limitations of this study: 1) Center A often performs laparoscopic mobilization of the splenic flexure, ligation of the inferior mesenteric artery and vein and entry into the TME plane, followed by a lower midline incision and completion of low anterior resections in patients with neoadjuvant therapy but codes this as open operation; 2) Center B had a higher fraction of transanal TME resections, which has been shown to result in higher complication rates at the beginning of the learning curve [26, 27].

Additionally, our study is of a retrospective nature and has respective limitations: the retrospective study design impairs identifying minor complications such as wound infections; To reduce the risk of not being able to accurately identify postoperative complications due to the retrospective nature of this study, dichotomization into major and minor complications was performed. Since all major complications (CDC > 2) require invasive treatments, all major complications must be documented and can therefore be confidently identified. Additionally, we did not separately analyze robotic surgery, due to the implementation of robotic programs and thus limited case volume during the study period. Moreover, we entered data from the electronic patient records that were logged at the time of surgery. These records did not account for teaching operations during the study period, but highly experienced surgeons are present and supervise rectal resections, and thus this limitation is unlikely to influence the overall outcome [28].

In conclusion, this work contributes valuable insights into the ongoing debate regarding the regionalization of surgery, especially CRC surgery in Austria. While the individual surgeon’s experience remains a crucial premise, we have shown that the center’s policy and set-up are of vital importance to the postoperative outcomes of CRC patients, irrespective of the operating surgeon. Future research should focus on implementing prospective real-time outcome monitoring in all hospitals offering colorectal resections to evaluate impact of individual and center’s caseloads on relevant outcome variables.

## Supplementary Information

Supplementary tables 1 and 2

## References

[1] Xie Y, Shi L, He X, Luo Y. Gastrointestinal cancers in China, the USA, and Europe. Gastroenterol Rep. 2021;9(2):91–104. 10.1093/gastro/goab010. 10.1093/gastro/goab010 PMC8128023 34026216

[2] Bray F, Ferlay J, Soerjomataram I, Siegel RL, Torre LA, Jemal A. Global cancer statistics 2018: GLOBOCAN estimates of incidence and mortality worldwide for 36 cancers in 185 countries. CA Cancer J Clin. 2018;68(6):394–424. 10.3322/caac.21492. 30207593 10.3322/caac.21492

[3] Tevis SE, Kennedy GD. Postoperative complications: looking forward to a safer future. Clin Colon Rectal Surg. 2016;29(3):246–52. 10.1055/s-0036-1584501. 27582650 10.1055/s-0036-1584501 PMC4991963

[4] Rawla P, Sunkara T, Barsouk A. Epidemiology of colorectal cancer: incidence, mortality, survival, and risk factors. Prz Gastroenterol. 2019;14(2):89–103. 10.5114/pg.2018.81072. 31616522 10.5114/pg.2018.81072 PMC6791134

[5] Ostenfeld EB, Nørgaard M, Thomsen RW, Iversen LH, Jacobsen JB, Søgaard M. Comorbidity and survival of Danish patients with colon and rectal cancer from 2000–2011: a population-based cohort study. Clin Epidemiol. 2013;5(Suppl 1):65–74. 10.2147/clep.S47154. 24227924 10.2147/CLEP.S47154 PMC3820479

[6] Flynn DE, Mao D, Yerkovich ST, Franz R, Iswariah H, Hughes A, et al. The impact of comorbidities on post-operative complications following colorectal cancer surgery. PLoS ONE. 2020;15(12):e243995. 10.1371/journal.pone.0243995. 33362234 10.1371/journal.pone.0243995 PMC7757883

[7] Cuthbert CA, Hemmelgarn BR, Xu Y, Cheung WY. The effect of comorbidities on outcomes in colorectal cancer survivors: a population-based cohort study. J Cancer Surviv. 2018;12(6):733–43. 10.1007/s11764-018-0710-z. 30191524 10.1007/s11764-018-0710-z

[8] Eppsteiner RW, Csikesz NG, McPhee JT, Tseng JF, Shah SA. Surgeon volume impacts hospital mortality for pancreatic resection. Ann Surg. 2009;249(4):635–40. 10.1097/SLA.0b013e31819ed958. 19300225 10.1097/SLA.0b013e31819ed958

[9] Birkmeyer JD, Stukel TA, Siewers AE, Goodney PP, Wennberg DE, Lucas FL. Surgeon volume and operative mortality in the United States. N Engl J Med. 2003;349(22):2117–27. 10.1056/NEJMsa035205. 14645640 10.1056/NEJMsa035205

[10] Wouters MW, Gooiker GA, van Sandick JW, Tollenaar RA. The volume-outcome relation in the surgical treatment of esophageal cancer: a systematic review and meta-analysis. Cancer. 2012;118(7):1754–63. 10.1002/cncr.26383. 22009562 10.1002/cncr.26383

[11] Nuttall M, van der Meulen J, Phillips N, Sharpin C, Gillatt D, McIntosh G, et al. A systematic review and critique of the literature relating hospital or surgeon volume to health outcomes for 3 urological cancer procedures. J Urol. 2004;172(6 Pt 1):2145–52. 10.1097/01.ju.0000140257.05714.45. 15538220 10.1097/01.ju.0000140257.05714.45

[12] Rogers SO Jr., Wolf RE, Zaslavsky AM, Wright WE, Ayanian JZ. Relation of surgeon and hospital volume to processes and outcomes of colorectal cancer surgery. Ann Surg. 2006;244(6):1003–11. 10.1097/01.sla.0000231759.10432.a7. 17122626 10.1097/01.sla.0000231759.10432.a7 PMC1856632

[13] Huo YR, Phan K, Morris DL, Liauw W. Systematic review and a meta-analysis of hospital and surgeon volume/outcome relationships in colorectal cancer surgery. J Gastrointest Oncol. 2017;8(3):534–46. 10.21037/jgo.2017.01.25. 28736640 10.21037/jgo.2017.01.25 PMC5506277

[14] Kressner M, Bohe M, Cedermark B, Dahlberg M, Damber L, Lindmark G, et al. The impact of hospital volume on surgical outcome in patients with rectal cancer. Dis Colon Rectum. 2009;52(9):1542–9. 10.1007/DCR.0b013e3181af58f4. 19690480 10.1007/DCR.0b013e3181af58f4

[15] Harling H, Bulow S, Moller LN, Jorgensen T, Danish Colorectal Cancer G. Hospital volume and outcome of rectal cancer surgery in Denmark 1994–99. Colorectal Dis. 2005;7(1):90–5. 10.1111/j.1463-1318.2004.00751.x. 15606594 10.1111/j.1463-1318.2004.00751.x

[16] Heald RJ, Moran BJ, Ryall RD, Sexton R, MacFarlane JK. Rectal cancer: the Basingstoke experience of total mesorectal excision, 1978–1997. Arch Surg. 1998;133(8):894–9. 10.1001/archsurg.133.8.894. 9711965 10.1001/archsurg.133.8.894

[17] Nica A, Sutradhar R, Kupets R, Covens A, Vicus D, Li Q, et al. Outcomes after the regionalization of care for high-grade endometrial cancers: a population-based study. Am J Obstet Gynecol. 2021;224(3):274 e1–274 e10. 10.1016/j.ajog.2020.09.012. 32931769 10.1016/j.ajog.2020.09.012

[18] Link KH, Coy P, Roitman M, Link C, Kornmann M, Staib L. Minimum volume discussion in the treatment of colon and rectal cancer: a review of the current status and relevance of surgeon and hospital volume regarding result quality and the impact on health economics. Visc Med. 2017;33(2):140–7. 10.1159/000456044. 28560230 10.1159/000456044 PMC5447170

[19] Clavien PA, Barkun J, de Oliveira ML, Vauthey JN, Dindo D, Schulick RD, et al. The Clavien-Dindo classification of surgical complications: five-year experience. Ann Surg. 2009;250(2):187–96. 10.1097/SLA.0b013e3181b13ca2. 19638912 10.1097/SLA.0b013e3181b13ca2

[20] NICE) NIfHaCE. NICE Guideline Colorectal Cancer. NICE Guideline. 2020:14.

[21] Yang J, Luo Y, Tian T, Dong P, Fu Z. Effects of neoadjuvant radiotherapy on postoperative complications in rectal cancer: a meta-analysis. J Oncol. 2022;2022:1–16. 10.1155/2022/8197701. 10.1155/2022/8197701 PMC8754670 35035483

[22] Schlager L, Monschein M, Schüller J, Bergmann M, Krall C, Razek P, et al. The predictive value of comorbidities on postoperative complication rates and overall survival in left-sided oncological colorectal resections: a multicentre cohort study. Int J Surg. 2023; 10.1097/JS9.0000000000000734. 37800585 10.1097/JS9.0000000000000734 PMC10720865

[23] Huang YM, Lee YW, Huang YJ, Wei PL. Comparison of clinical outcomes between laparoscopic and open surgery for left-sided colon cancer: a nationwide population-based study. Sci Rep. 2020;10(1):75. 10.1038/s41598-019-57059-6. 31919417 10.1038/s41598-019-57059-6 PMC6952445

[24] van Gijn W, Gooiker GA, Wouters MW, Post PN, Tollenaar RA, van de Velde CJ. Volume and outcome in colorectal cancer surgery. Eur J Surg Oncol. 2010;36(Suppl 1):S55–S63. 10.1016/j.ejso.2010.06.027. 20615649 10.1016/j.ejso.2010.06.027

[25] Borowski DW, Bradburn DM, Mills SJ, Bharathan B, Wilson RG, Ratcliffe AA, et al. Volume-outcome analysis of colorectal cancer-related outcomes. Br J Surg. 2010;97(9):1416–30. 10.1002/bjs.7111. 20632311 10.1002/bjs.7111

[26] Vannijvel M, Wolthuis AM. Limitations and concerns with transanal total mesorectal excision for rectal cancer. Clin Colon Rectal Surg. 2022;35(2):141–5. 10.1055/s-0041-1742115. 35237110 10.1055/s-0041-1742115 PMC8885157

[27] Penna M, Hompes R, Mackenzie H, Carter F, Francis NK. First international training and assessment consensus workshop on transanal total mesorectal excision (taTME). Tech Coloproctol. 2016;20(6):343–52. 10.1007/s10151-016-1454-2. 27015679 10.1007/s10151-016-1454-2

[28] Bhoday J, Martling A, Strassburg J, Brown G. Session 1: the surgeon as a prognostic factor in colon and rectal cancer? Colorectal Dis. 2018;20(Suppl 1):36–8. 10.1111/codi.14076. 29878669 10.1111/codi.14076

