# Supplementary material for: Does surgeon or hospital volume influence outcome in dedicated colorectal units?—A Viennese perspective
Source: Wien Klin Wochenschr. 2024 Aug 2;137(7-8):231–6. doi: 10.1007/s00508-024-02405-6 (PMC12006224; doi:10.1007/s00508-024-02405-6)
Supplement: Supplementary file 1 — Supplementary tables 1 and 2 [file 508_2024_2405_MOESM1_ESM.docx]

Supp. Table 1

|  | | **Center A** | **Center B** |
| --- | --- | --- | --- |
| **Sex** | **Male (*%*)** | 175 (*67.6*) | 52 (*67.5*) |
|  | **Female (*%*)** | 84 (*32.4*) | 25 (*32.5*) |
| **Active Smoking Status** | **Yes (*%*)** | 213 (*82.2*) | 62 (*80.5*) |
|  | **No (*%*)** | 46 (*17.8*) | 15 (*19.5*) |
| **CCI** | **High (*%*)** | 138 (*53.3*) | 40 (*51.9*) |
|  | **Low (*%*)** | 121 (*46.7*) | 37 (*48.1*) |
| **BMI** | **Mean (*Standard Deviation*)** | 25.2 (*4.2*) | 25.7 (*4.4*) |
| **Diabetes Mellitus** | **Yes (*%*)** | 218 (*84.2*) | 65 (*84.4*) |
|  | **No (*%*)** | 32 (*12.4*) | 12 (*15.6*) |
| **Indication** | **Elective** (*%*) | 259 (*100.0*) | 77 (*100.0*) |
|  | **Emergency** (*%*) | 0 (*0.0*) | 0 (*0.0*) |
| **OP-Type** | **Minimally invasive surgery (*%*)** | 124 (*47.9*) | 75 (*97.4*) |
|  | **Open surgery (*%*)** | 135 (*52.1*) | 2 (*2.6*) |
| **Performed Operation** | **Low Anterior Resection (*%*)** | 208 (*80.3*) | 45 (*58.4*) |
|  | **TaTME (*%*)** | 5 (*1.9*) | 19 (*24.7*) |
|  | **AP (*%*)** | 42 (*16.2*) | 12 (*15.6*) |
|  | **Others (*%*)** | 0 (*0*) | 1 (*1.3*) |
| **Resection Margins** | **R0 (*%*)** | 252 (*97.3*) | 70 (*90.9*) |
|  | **R1 or R2 (*%*)** | 7 (*2.7*) | 7 (*9.1*) |
| **Number of positive Lymph Nodes** | **Mean (*Standard Deviation)*** | 1.5 (*3.6*) | 1.1 (*2.7*) |
| **T-Stage*** | **1 (*%*)** | 25 (*9.7*) | 12 (*15.6*) |
|  | **2 (*%*)** | 67 (*25.9*) | 14 (*18.2*) |
|  | **3 (*%*)** | 129 (*49.8*) | 45 (*58.4*) |
|  | **4 (*%*)** | 19 (*7.3*) | 3 (*3.9*) |
| **N-Stage*** | **0 (%)** | 157 (*60.6*) | 51 (*66.2*) |
|  | **1 (%)** | 68 (*26.3*) | 17 (*22.1*) |
|  | **2 (%)** | 34 (*13.2*) | 9 (*11.7*) |
| **M-Stage** | **0 (%)** | 214 (*82.9*) | 66 (*85.7*) |
|  | **1 (%)** | 44 (*17.1*) | 11 (*14.3*) |
| **Neoadjuvant Therapy** | **None (*%*)** | 115 (*44.6*) | 32 (*36.3*) |
|  | **Chemoradiotherapy (*%*)** | 114 (*44.2*) | 44 (*57.1*) |
|  | **Radiotherapy (*%*)** | 24 (*9.3*) | 1 (*1.3*) |
|  | **Others (*%*)** | 5 (*1.9*) | 0 (*0*) |
| **Adjuvant Therapy** | **None (*%*)** | 209 (*80.7*) | 32 (*41.6*) |
|  | **Yes (*%*)** | 50 (*19.3*) | 45 (*58.4*) |
| **Tumor Localisation** | **Rectosigmoid Junction (*%*)** | 14 (*6.0*) | 0 (*0*) |
|  | **Upper Rectum (*%*)** | 23 (*9.8*) | 19 (*24.7*) |
|  | **Middle Rectum (*%*)** | 52 (*22.1*) | 28 (*36.4*) |
|  | **Lower Rectum (*%*)** | 142 (*60.4*) | 30 (*39.0*) |
|  | **Anus (*%*)** | 2 (*0.9*) | 0 (*0*) |
| **Distance from anorectal junction to tumor in cm^§^** | **Mean (*Standard Deviation*)** | 7.5 (*4.3)* | 7.6 (*4.6*) |
| **Rehospitalisation within 90 days** | **Yes (*%*)** | 40 (*15.4)* | 18 (*23.4*) |
|  | **No (*%*)** | 219 (*84.6*) | 59 (*76.6*) |
| **Clavien Dindo Score** | **Low (*%*)** | 217 (*83.8*) | 48 (*62.3*) |
|  | **High (*%*)** | 42 (16.2) | 29 (*37.7*) |

*Supp. Table 1* – Similar distribution of patients regarding the baseline characteristics sex, active smoking status, and CCI between the centers. *T and N stage is reported as per final pathological report. **^§^**Measured on pre-operative MRI.

Abbreviations: CCI = Charlson Comorbidity Index; BMI = Body Mass Index; TaTME = Transanal Total Mesorectal Excision; AP = Abdominoperineal Resection

Supp. Table 2

|  | | **Low volume surgeons** | | **High volume surgeons** | |
| --- | --- | --- | --- | --- | --- |
| **Sex** | **Male** (*%*) | 75 (*66.4*) | | 152 (*68.5*) | |
|  | **Female** (*%*) | 38 (*33.6*) | | 70 (*31.5*) | |
| **Active Smoking Status** | **Yes** (*%*) | 25 (*22.1*) | | 36 (*16.2*) | |
|  | **No** (*%*) | 88 (*77.9*) | | 186 (*83.6*) | |
| **CCI** | **High** (*%*) | 60 (*53.1*) | | 98 (*44.1*) | |
|  | **Low** (*%*) | 53 (*46.9*) | | 124 (*55.9*) | |
| **BMI** | **Mean** (*Standard Deviation*) | 25.8 (*4.2*) | | 25.3 (*4.4*) | |
| **Diabetes Mellitus** | **Yes** (*%*) | 20 (*17.7*) | | 24 (*10.8*) | |
|  | **No** (*%*) | 92 (*81.4*) | | 190 (*85.6*) | |
| **Indication** | **Elective** (*%*) | 113 (*100.0*) | | 222 (*100.0*) | |
|  | **Emergency** (*%*) | 0 (*0.0*) | | 0 (*0.0*) | |
| **OP-Type** | **Minimally invasive Surgery** (*%*) | 60 (*53.1*) | | 139 (*62.6*) | |
|  | **Open** (*%*) | 53 (*46.9*) | | 83 (*37.4*) | |
|  | **Low Anterior Resection** (*%*) | 85 (*75.2*) | | 168 (*75.7*) | |
|  | **TaTME** (*%*) | 3 (*2.7*) | | 21 (*9.5*) | |
|  | **AP** (*%*) | 23 (*20.4*) | | 31 (*14.0*) | |
|  | **Others** (*%*) | 1 (*0.9*) | | 0 (*0.0*) | |
| **Resection Margins** | **R0** (*%*) | 109 (*96.5*) | | 212 (*95.5*) | |
|  | **R1 or R2** (*%*) | 4 (*3.5*) | | 10 (*4.5*) | |
| **Number of positive Lymph Nodes** | **Mean** (*Standard Deviation)* | 1.7 (*3.7*) | | 1.3 (*3.1*) | |
| **T-Stage** | **1** (*%*) | 11 (*9.7*) | | 26 (*11.7*) | |
|  | **2** (*%*) | 27 (*23.9*) | | 54 (*24.3*) | |
|  | **3** (*%*) | 60 (*53.1*) | | 114 (*51.4*) | |
|  | **4** (*%*) | 6 (*5.4*) | | 16 (*7.3*) | |
| **N-Stage** | **0** (%) | 64 (*56.6*) | | 143 (*64.4*) | |
|  | **1** (%) | 31 (*27.5*) | | 54 (*24.4*) | |
|  | **2** (%) | 18 (*15.9*) | | 25 (*11.3*) | |
| **M-Stage** | **0** (%) | 85 (*75.2*) | | 194 (*87.8*) | |
|  | **1** (%) | 28 (*24.8*) | | 27 (*12.2*) | |
| **Neoadjuvant Therapy** | **None** (*%*) | 46 (*40.7*) | | 101 (*45.7*) | |
|  | **Chemoradiotherapy** (*%*) | *56* (*49.6*) | | 101 (*45.7*) | |
|  | **Radiotherapy** (*%*) | 10 (*8.8*) | | 15 (*6.8*) | |
|  | **Others** (*%*) | 1 (*0.9*) | | 4 (*1.8*) | |
| **Adjuvant Therapy** | **None** (*%*) | 83 (*73.5*) | | 157 (*70.7*) | |
|  | **Yes** (*%*) | 30 (*26.5*) | | 65 (*29.3*) | |
| **Tumor Localisation** | **Rectosigmoid Junction** (*%*) | 2 (*2.0*) | | 12 (*5.7*) | |
|  | **Upper** **Rectum** (*%*) | 15 (*14.7*) | | 27 (*12.9*) | |
|  | **Middle** **Rectum** (*%*) | 21 (*20.6*) | | 59 (*28.2*) | |
|  | **Lower** **Rectum** (*%*) | 60 (*58.8*) | | 111 (*53.1*) | |
|  | **Anus** (*%*) | 2 (*2.0*) | | 0 (*0.0*) | |
| **Distance from anorectal junction to tumor in cm ^§^** | **Mean** (*Standard Deviation*) | *7.6* (*4.4*) | | 7.5 (*4.4*) | |
| **Rehospitalisation within 90 days** | **Yes** (*%*) | 17 (*15*) | | 41 (*18.5*) | |
|  | **No** (*%*) | 96 (*85*) | | 181 (*81.5*) | |
| **Clavien Dindo Score** | **Low (*%*)** | | 93 (*82.3*) | | 171 (*77.0*) |
|  | **High (*%*)** | | 20 (*17.7*) | | 51 (*23.0*) |

*Supp. Table 2* – Similar distribution of patients regarding the baseline characteristics sex, active smoking status, and CCI between the different surgeon volume groups. *T and N stage is reported as per final pathological report. **^§^**Measured on pre-operative MRI.

Abbreviations: CCI = Charlson Comorbidity Index; BMI = Body Mass Index; TaTME = Transanal Total Mesorectal Excision; AP = Abdominoperineal Resection
